# The WHO Bacterial Priority Pathogens List 2024: a prioritisation study to guide research, development, and public health strategies against antimicrobial resistance

**DOI:** 10.1016/S1473-3099(25)00118-5

**Published:** 2025-09

**Authors:** Hatim Sati, Elena Carrara, Alessia Savoldi, Paul Hansen, Jacopo Garlasco, Enrica Campagnaro, Simone Boccia, Juan Antonio Castillo-Polo, Eugenia Magrini, Pilar Garcia-Vello, Eve Wool, Valeria Gigante, Erin Duffy, Alessandro Cassini, Benedikt Huttner, Pilar Ramon Pardo, Mohsen Naghavi, Fuad Mirzayev, Matteo Zignol, Alexandra Cameron, Evelina Tacconelli, WHO Bacterial Priority Pathogens List Advisory Group, Aaron Aboderin, Majed Al Ghoribi, Jameela Al-Salman, Afreenish Amir, Anucha Apisarnthanarak, Martin Blaser, Amany El-Sharif, Sabiha Essack, Stephan Harbarth, Xun Huang, Geetanjali Kapoor, Gwenan Knight, Jeremiah Chakaya Muhwa, Dominique L. Monnet, Timothée Ousassa, Rosa Sacsaquispe, Juliëtte Severin, Motoyuki Sugai, Neelam Taneja, Alaine Umubyeyi Nyaruhirira

**Affiliations:** a Impact Initiatives and Research Coordination Unit, Division of Antimicrobial Resistance, World Health Organization, Geneva, Switzerland; b Division of Infectious Diseases, Department of Diagnostic and Public Health, University of Verona, Verona, Italy; c Department of Economics, University of Otago, Dunedin, New Zealand; d Institute for Health Metrics and Evaluation, University of Washington, Seattle, WA, USA; e Combating Antibiotic-Resistant Bacteria Biopharmaceutical Accelerator, Boston, MA, USA; f Infectious Diseases Service, Lausanne Université Hospital, Lausanne, Switzerland; g Department of Surveillance, Prevention and Control, Division of Antimicrobial Resistance, World Health Organization, Geneva, Switzerland; h Antimicrobial Resistance Special Program, Regional Office for the Americas of the World Health Organization, Washington, DC, USA; i World Health Organization Global Tuberculosis Programme, Geneva, Switzerland

## Abstract

**Background:**

The 2017 WHO Bacterial Priority Pathogens List (BPPL) has been instrumental in guiding global policy, research and development, and investments to address the most urgent threats from antibiotic-resistant pathogens, and it is a key public health tool for the prevention and control of antimicrobial resistance (AMR). Since its release, at least 13 new antibiotics targeting bacterial priority pathogens have been approved. The 2024 WHO BPPL aims to refine and build on the previous list by incorporating new data and evidence, addressing previous limitations, and improving pathogen prioritisation to better guide global efforts in combating AMR.

**Methods:**

The 2024 WHO BPPL followed a similar approach to the first prioritisation exercise, using a multicriteria decision analysis framework. 24 antibiotic-resistant bacterial pathogens were scored based on eight criteria, including mortality, non-fatal burden, incidence, 10-year resistance trends, preventability, transmissibility, treatability, and antibacterial pipeline status. Pathogens were assessed on each of the criteria on the basis of available evidence and expert judgement. A preferences survey using a pairwise comparison was administered to 100 international experts (among whom 79 responded and 78 completed the survey) to determine the relative weights of the criteria. Applying these weights, the final ranking of pathogens was determined by calculating a total score in the range of 0–100% for each pathogen. Subgroup and sensitivity analyses were conducted to assess the impact of experts’ consistency, background, and geographical origin on the stability of the rankings. An independent advisory group reviewed the final list, and pathogens were subsequently streamlined and grouped into three priority tiers based on a quartile scoring system: critical (highest quartile), high (middle quartiles), and medium (lowest quartile).

**Findings:**

The pathogens’ total scores ranged from 84% for the top-ranked bacterium (carbapenem-resistant *Klebsiella pneumoniae*) to 28% for the bottom-ranked bacterium (penicillin-resistant group B streptococci). Antibiotic-resistant Gram-negative bacteria (including *K pneumoniae, Acinetobacter* spp, and *Escherichia coli*), as well as rifampicin-resistant *Mycobacterium tuberculosis*, were ranked in the highest quartile. Among the bacteria commonly responsible for community-acquired infections, the highest rankings were for fluoroquinolone-resistant *Salmonella enterica* serotype Typhi (72%), *Shigella* spp (70%), and *Neisseria gonorrhoeae* (64%). Other important pathogens on the list include *Pseudomonas aeruginosa* and *Staphylococcus aureus*. The results of the preferences survey showed a strong inter-rater agreement, with Spearman's rank correlation coefficient and Kendall's coefficient of concordance both at 0·9. The final ranking showed high stability, with clustering of the pathogens based on experts’ backgrounds and origins not resulting in any substantial changes to the ranking.

**Interpretation:**

The 2024 WHO BPPL is a key tool for prioritising research and development investments and informing global public health policies to combat AMR. Gram-negative bacteria and rifampicin-resistant *M tuberculosis* remain critical priority pathogens, underscoring their persistent threat and the limitations of the current antibacterial pipeline. Focused efforts and sustained investments in novel antibacterials are needed to address AMR priority pathogens, which include high-burden antibiotic-resistant bacteria such as *Salmonella* and *Shigella* spp, *N gonorrhoeae*, and *S aureus*. Beyond research and development, efforts to address these pathogens should also include expanding equitable access to existing drugs, enhancing vaccine coverage, and strengthening infection prevention and control measures.

**Funding:**

This work is based on the development of the 2024 WHO BPPL, which was conducted by the WHO AMR Division through grants from the Government of Austria, the Government of Germany, the Government of Saudi Arabia, and the European Commission's Health Emergency Preparedness and Response Authority.

**Translations:**

For the Arabic, French, Italian, Japanese and Spanish translations of the abstract see Supplementary Materials section.

## Introduction

The emergence and spread of antimicrobial resistance (AMR) threaten the effective prevention and treatment of an ever-increasing range of infections caused by bacteria, parasites, viruses, and fungi.^1^, ^2^ Among all AMR pathogens, antibiotic-resistant pathogens are associated with the highest burden and health-care costs.^3^ In 2019, antibiotic-resistant infections caused an estimated 1·27 million deaths (95% CI 0·86–1·91) globally, disproportionately affecting low-income and middle-income countries.^3^ A systematic analysis estimated that 4·71 million deaths (95% uncertainty interval 4·23–5·19) were associated with bacterial AMR in 2021, including 1·14 million deaths (1·00–1·28) attributable to bacterial AMR.^4^

In response to the AMR threat, WHO introduced the Global Action Plan on Antimicrobial Resistance in 2015.^5^ Subsequently WHO launched its first Bacterial Priority Pathogens List (BPPL) in 2017,^6^ which had the aim of guiding the research and development of new antibacterials and was established based on pathogen resistance profiles, public health impact, and the need for new treatments. This list featured 25 antibiotic-resistant pathogens streamlined into 13 families across three priority tiers: critical, high, and medium.^6^, ^7^ Since its development, the WHO BPPL has directed research and development efforts toward pressing AMR challenges,^8^ and has guided AMR surveillance and other prevention and control efforts.^9^, ^10^

**Research in context**

**Evidence before this study**

Antimicrobial resistance (AMR), largely fuelled by antibiotic resistance, is an escalating global health threat. WHO has long recognised this challenge, notably through the 2015 WHO Global Action Plan on Antimicrobial Resistance and the development of the 2017 WHO Bacterial Priority Pathogens List (BPPL), which has since provided a key framework for guiding research and development, AMR surveillance, and public health strategies. Since the release of the 2017 BPPL, 13 new antibiotics have been authorised. However, AMR continues to emerge, with many pathogens now showing resistance to most newer antibiotics. As AMR evolves, so too must global understanding and priorities. Since 2017, new evidence and data have become available, deepening our knowledge of AMR trends and the shifting landscape of resistance. This ongoing progression necessitates an update to the original BPPL to better address the current challenges posed by resistant bacteria. This study presents the updated 2024 WHO BPPL, which addresses limitations of the 2017 WHO BPPL and incorporates the latest global data on AMR. To understand the existing evidence before this study and inform this update, we conducted a targeted literature search of PubMed and Google Scholar for studies published between Jan 1, 2017, and Nov 30, 2022, using the following search terms: “priority list AND infections”, “priority list AND resistance”, and “R&D AND priority AND bacteria”, combined with “antibiotic AND priority AND infections OR bacteria”. No limitations were placed on the type of publication or language. This search identified seven relevant publications, including the 2017 WHO BPPL report, a systematic analysis of the global burden of bacterial AMR infections in 2019, and five national priority pathogen lists. These publications informed the preliminary selection of bacterial pathogens for this prioritisation study.

**Added value of this study**

Building on the methodology and data used in the development of the 2017 WHO BPPL, the 2024 WHO BPPL introduces substantial revisions, leveraging more robust quantitative data to evaluate pathogens based on disease burden, resistance trends, and public health impact. It also incorporates qualitative criteria, such as preventability, transmission dynamics, and treatability, emphasising disparities in pathogen burden and access to treatment between high-income countries and low-income and middle-income countries. Compared with the 2017 iteration, the list introduces additional pathogens and reclassifies others, now emphasising the disproportionate burden of community-acquired infections in resource-limited settings.

**Implications of the evidence**

The 2024 BPPL underscores the persistent threat of antibiotic-resistant Gram-negative bacteria and emerging resistance to newer antibiotics. The list introduces additional pathogens andreclassifies others, emphasising the disproportionate burden ofcommunity-acquired infections (eg, rifampicin-resistant*M tuberculosis* and fluroquinolone-resistant *Salmonella* and*Shigella* spp) in low-income and middle-income countries. The findings reinforce the urgent need for sustained research and development investments, international collaboration, and multifaceted interventions, including new antibiotics, vaccines, enhanced surveillance, infection prevention, and expanded water, sanitation, and hygiene initiatives, particularly in resource-limited settings. The 2024 BPPL highlights the need for innovation—not only in drug development but also in diagnostics, treatment strategies, and scalable public health solutions—to combat AMR effectively. This updated list provides a robust, evidence-based framework to guide and prioritise global efforts against AMR.

In the 7 years since the release of the initial WHO BPPL, 13 new antibiotics targeting bacterial priority pathogens have been authorised, many of which have been listed in the WHO Essential Medicines List.^8^, ^11^ Additionally, pretomanid, which was approved in 2019, has been recommended by WHO as part of a novel regimen to treat multidrug-resistant and rifampicin-resistant *Mycobacterium tuberculosis*.^12^

Despite this progress, AMR continues to evolve, with complex resistance patterns emerging, including resistance to most newer antibiotics.^13^, ^14^ Concerningly, investments in research and development, prevention, and control remain insufficient.

To ensure ongoing impact and relevance, WHO has developed an updated BPPL that integrates the latest data on AMR, addresses limitations from the 2017 list, and considers the broader public health impacts of pathogens.^15^ This Article outlines the methodology and results of the 2024 update of the WHO BPPL, discussing implications, study limitations, and proposed directions for future AMR research, interventions, and policy.

## Methods

### Expert selection

Two expert groups participated in the update. The first group, the WHO BPPL Advisory Group, was selected through an open call for experts conducted from July 16 to Sept 1, 2021.^16^ From 120 applications received, 23 experts were selected, representing all six WHO regions. The group size was limited to less than 25 per WHO guidance, to facilitate more effective consensus-building and conflict management, with additional input from observers and a second survey group. The selection process ensured gender balance, geographical diversity, and a range of professional backgrounds and expertise, with regular conflict-of-interest assessments conducted in line with WHO standards. These experts were involved in every stage of the process, including protocol updates, pathogen inclusion and exclusion decisions, criteria definitions and selection, and pathogen ratings based on the established criteria.

The second group comprised 100 AMR experts, including the 23 WHO BPPL Advisory Group members. The additional experts were invited to ensure an adequate number of participants, diverse disciplinary backgrounds, and balanced geographical and gender representation for the final survey. This broader group completed the preferences survey used to determine the relative weights of the criteria.

### Study design

The update followed the same multicriteria decision analysis (MCDA) approach used in 2017.^6^ MCDA is a scientific decision-making method that evaluates alternatives based on multiple criteria, enabling systematic and transparent decision making and regular updates as new evidence or threats emerge. A key advantage of the method is that it integrates quantitative evidence and expert judgement to evaluate and rank alternatives in complex decision-making situations. The process involved four main steps: (1) selecting the bacterial pathogens to be ranked and criteria to assess them; (2) synthesising evidence to rate each pathogen against the criteria selected; (3) conducting a preferences survey to weight criteria and compute rankings; and (4) performing stability assessments through subgroup and sensitivity analyses.^6^, ^7^

### Pathogen selection

To understand the existing evidence before this study and inform the selection of the pathogens for the update, we conducted a targeted literature search of PubMed and Google Scholar from Jan 1, 2017, to Nov 30, 2022, using the following search terms: “priority list AND infections”, “priority list AND resistance”, and “R&D AND priority AND bacteria”, combined with “antibiotic AND priority AND infections OR bacteria”. No limitations were placed on the type of publication or language. This search identified seven relevant publications, including the 2017 WHO BPPL, a 2019 systematic analysis of the global burden of AMR, and five national priority pathogen lists. These publications informed the preliminary selection of bacterial pathogens for this prioritisation study.

Consensus on the pathogens to prioritise was achieved using a modified Delphi approach, involving a survey administered to the BPPL Advisory Group experts via the REDCap application and informed by expert discussions.^17^ Five pathogens from the 2017 WHO BPPL were removed based on evidence and consensus: clarithromycin-resistant *Helicobacter pylori*, fluoroquinolone-resistant *Campylobacter* spp, penicillin-non-susceptible *Streptococcus pneumoniae*, third-generation cephalosporin-resistant *Providencia* spp, and vancomycin-intermediate and vancomycin-resistant *Staphylococcus aureus*. Four new combinations were added to the updated list, including rifampicin-resistant *M tuberculosis*, resulting in 24 antibiotic-resistance phenotypes.

### Development of assessment criteria

In 2017, ten criteria were selected to assess and rank the pathogens, adhering to MCDA best practices for criteria selection, which emphasise completeness, non-redundancy, and preference independence.^6^ These criteria comprised three quantitative measures—mortality, prevalence, and 10-year resistance trends—and seven qualitative measures, including health-care and community burden, preventability, and treatability.^6^ For the 2024 update, the criteria were substantially revised and streamlined into eight measures to address gaps, incorporate lessons learned, and integrate new data.^15^ Quantitative measures were expanded to include mortality (case-fatality ratios), years lived with disability (YLD), incidence, and 10-year resistance trends, reflecting the availability of more granular and reliable data on both fatal and non-fatal AMR disease burdens. The definitions and scoring indices for the qualitative criteria—preventability, transmissibility, treatability, and antibacterial pipeline status—were also revised to enhance consistency and rigour in their application. These refinements were finalised through a collaborative process involving a WHO steering group and the WHO BPPL Advisory Group, with expert opinion supplementing literature-based evidence where necessary.^15^ Table 1 provides a summary of these criteria, their definitions, scoring systems, and levels.

**Table 1:** Criteria definitions and scoring systems

		**Description**
**Mortality**		
Definition		Case-fatality ratio (%)—ie, the pooled prevalence of all-cause mortality among patients with infections caused by antibiotic-resistant bacteria
Scoring system		
	High	>30%
	Medium-high	21–30%
	Medium	11–20%
	Medium-low	5–10%
	Low	<5%
**Incidence**		
Definition		Global incidence of cases of infection caused by antibiotic-resistant bacteria per million population (all ages, all sexes)
Scoring system		
	High	>10 000 cases per million population
	Medium-High	5001–10 000 cases per million population
	Medium	1001–5000 cases per million population
	Medium-Low	100–1000 cases per million population
	Low	<100 cases per million population
**Non-fatal health burden**		
Definition		YLDs per million population, including all ages and all sexes, attributable to infections by antibiotic-resistant bacteria
Scoring system		
	High	>1·5 YLDs per million population
	Medium-High	1·1–1·5 YLDs per million population
	Medium	0·51–1 YLDs per million population
	Medium-Low	0·11–0·5 YLDs per million population
	Low	≤0·1 YLDs per million population
**Trends in resistance**		
Definition		10-year trend in resistance rate, defined as the percentage of antibiotic-resistant bacteria out of the total number of isolates tested
Scoring system		
	Level 5	Increasing trend in three or more WHO regions (or in most regions with data)
	Level 4	Increasing trend in two WHO regions
	Level 3	Increasing trend in one WHO region
	Level 2	Stable trend in all WHO regions
	Level 1	Significantly decreasing trend in at least one WHO region, with no increase in the others
**Transmissibility**		
Definition		Evidence of transmission of the antibiotic-resistant bacteria among different pathways in two distinct domains: (1) outbreak capability (human-to-human transmission in health-care or community settings); and (2) transmission potential between humans and animal, food, and environmental compartments
Scoring system		
	High	Well documented outbreak capability and high transmission potential (defined as the capability of spreading between humans and across other One Health compartments)
	Medium-high	Well documented outbreak capability and moderate transmission potential; or moderately documented outbreak capability and high transmission potential
	Medium	Poorly documented outbreak capability and high transmission potential;well documented outbreak capability and low transmission potential; or moderately documented outbreak capability and moderate transmission potential
	Medium-low	Moderately documented outbreak capability and low transmission potential; or poorly documented outbreak capability and moderate transmission potential
	Low	Poorly documented outbreak capability and low transmission potential
**Preventability**		
Definition		The existence and effectiveness of preventive measures in containing the transmission of the antibiotic-resistant bacteria and reducing disease burden according to two distinct aspects of preventability: (1) individual-based infection prevention and control measures (including hand hygiene and standard and transmission-based precautions); and (2) community-based infection prevention and control measures (including vaccination, water, sanitation, and hygiene, access to health services, and food safety)
Scoring system		
	High	>5 points
	Medium-high	5 points
	Medium	4 points
	Medium-low	3 points
	Low	<3 points
Scoring criteria		
	Infection prevention and control measures	Effective and sufficient (2 points); recommended, existing, and effective (1 point); or not universally recommended due to low efficacy or feasibility (0 points)
	Decolonisation or chemoprophylaxis	Existing and effective (2 points); existing and partly effective or restricted to patients at high risk (1 point); or not existing or ineffective (0 points)
	Public health interventions in the community	Existing and effective, or not needed (2 points); existing and partly effective (1 point); or not existing or ineffective (0 points)
**Treatability**		
Definition		Composite criterion encompassing number of molecules listed in the guidelines, their efficacy ranking (first or lower lines of treatment *vs* last resort), safety profile, availability of oral or OPAT formulation, presence of paediatric formulation, concomitant resistance, and cost
Scoring system		
	High	>12 points
	Medium-high	10–11 points
	Medium	8–9 points
	Medium-low	6–7 points
	Low	≤5 points
Scoring criteria		
	Number of first-line options recommended by evidence-based guidelines	One antibiotic class (2 points); or two or more antibiotic classes (2 points per option)
	Concomitant resistance reported for first-line option	>20% (−1 point per option); or ≤20% (0 points)
	Availability of alternative options for the most typical infectious syndrome	No option available, or options available but with a poor toxicity profile, or option available but recommended only in combination (−1 point); options available with a fair toxicity profile, recommended in monotherapy, but with co-resistance >20% (0 points); or at least one alternative available with a fair toxicity profile, recommended also in monotherapy, and with co-resistance ≤20% (1 point)
	Formulations	Availability of oral options (1 point); availability of OPAT option (1 point); or available options approved or tested for paediatric population (1 point)
	Accessibility	High cost (−1 point); or low cost (0 points)
**Antibacterial pipeline**		
Definition		Extent to which the antibacterial pipeline, both currently and over the next 5–7 years, can effectively meet the clinical needs for treating each antibiotic-resistant bacterium; the criterion considers the number of newly approved antibiotics in the last 5–7 years, as well as the number of candidates in the clinical developmental pipeline that meet WHO innovation criteria (such as new chemical classes, novel targets, and absence of cross-resistance), and it also evaluates the availability of oral formulations for both the new candidates and those under development
Scoring system*		
	Unlikely	The pathogen has few or no potential active candidates in phase 1–3 according to WHO clinical pipeline analyses from July, 2017 to Nov, 2021; the pathogen has few or no candidates with ongoing MAAs or NDAs; or the pathogen had very few or no newly approved antibiotics between July, 2017 and Dec, 2022
	Possible	The pathogen has one or more potential active candidates in phase 1–3 according to WHO clinical pipeline analyses from July, 2017 to Nov, 2021; the pathogen has one or more candidates with ongoing MAAs or NDAs; or the pathogen had one or more newly approved antibiotics between July, 2017 and Dec, 2022
	Likely	The pathogen has a robust pipeline with multiple potential active candidates in phase 1–3 according to WHO clinical pipeline analyses from July, 2017 to Nov, 2021; or the pathogen has multiple candidates with ongoing MAAs or NDAs

The levels for each criterion were determined using a combination of quantitative and qualitative evidence. Quantitative criteria (ie, mortality, incidence, non-fatal health burden, and trends in resistance) were defined using numerical scaling based on thresholds derived from a comprehensive review of available empirical literature and data. Qualitative criteria (ie, transmissibility, preventability, treatability, and antibacterial pipeline) were defined using ordinal scales and assessed through scoring indices. These assessments were informed by available evidence and, when needed, supplemented by expert consensus. The relative weights of these criteria were subsequently defined through a PAPRIKA survey, ensuring a systematic and participatory approach to prioritisation. Table adapted from the 2024 WHO BPPL report.^15^ BPPL= Bacterial Priority Pathogens List. MAA=market authorisation application. NDA=new drug application. OPAT=outpatient parenteral antibiotic therapy. PAPRIKA=Potentially All Pairwise Rankings of All Possible Alternatives. YLDs=years lived with disability.

* Points were assigned according to a scoring system based on number of newly approved antibiotics (2017–22), number and novelty of pipeline candidates (based on WHO innovation criteria), and availability of oral formulations; point totals were used to assign pathogens to the categories of unlikely, possible, or likely based on predefined thresholds, as described in the WHO BPPL report.^15^

### Evidence and data synthesis

In line with the MCDA approach, evidence was reviewed to evaluate the 24 pathogens on the eight defined criteria. The main data sources included systematic literature reviews (for mortality and transmissibility criteria), the empirical data used in 2019 and 2021 systematic analyses of the global burden of AMR (for incidence and non-fatal burden),^3^, ^4^ 23 national and international surveillance systems (for the 10-year AMR trends), 91 treatment and prevention guidelines (for preventability and treatability),^12^, ^15^ and data from the WHO analysis of the antibacterial pipeline.^8^ Quantitative data were synthesised through partially Bayesian multilevel models, considering the heterogeneity between countries (when possible with available data), or alternatively through meta-analytical pooling of prevalence data and weighted logistic regression, with subgroup analyses by country income (World Bank income level) and WHO region. Statistical analyses were done using R software (version 4.2.2). Qualitative criteria ratings were initially informed by literature reviews and subsequently finalised through expert discussion and consensus by the WHO BPPL Advisory Group. This process aimed to address major evidence gaps with expert input. Details on data sources, a summary of the data synthesis, and data gaps are available in the WHO BPPL 2024 report.^15^ Examples of bacterial pathogen assessments conducted using the MCDA approach, based on the eight criteria, are summarised in the appendix.

### Determination of criteria weights (preferences survey)

A blinded preferences survey employing the Potentially All Pairwise Rankings of All Possible Alternatives (PAPRIKA) method was used to determine the criteria weights, similar to the approach taken in the 2017 WHO BPPL.^7^, ^18^ The survey was sent to 100 experts across all six WHO regions, ensuring diversity in geography, gender, and expertise. Survey creation, data collection, distribution, and analysis were supported by the dedicated MCDA software 1000minds. Based on the experts’ responses, the software attributed a different weight to each criterion, allowing pathogens to be scored on a range from 0% to 100%. Subgroup analyses by participants’ expertise, geographical origin, and country income level were also done using the same software. Two repeated questions were presented to each participant during the survey to assess their consistency.

### Final list categorisation and presentation

Pathogens scoring above the 75th percentile were classified as critical, those between the 25th and 75th percentiles as high, and those below the 25th percentile as medium. To streamline the presentation of results, pathogens with multiple resistance patterns within the same species or order were consolidated and ranked based on the highest position of any pathogen within that species or order. For example, different carbapenem-resistant Enterobacterales ranked first, fifth, and twelfth were grouped and assigned to the first position.

### Ranking stability assessment

Spearman's rank correlation and Kendall's coefficient of concordance were used to assess agreement among participants and to evaluate variations in the relative importance of the criteria and bacterium ranking due to differences in participants’ backgrounds. A sensitivity analysis evaluated consistency, expertise, country income, and geographical location to identify potential variations in weights assigned to each criterion. The statistical significance of such differences between groups was assessed using a MANOVA and post-hoc Tukey's differences.^19^ p<0·05 was considered significant for all analyses.

### Role of the funding source

The funders of the study had no role in the study design, data collection, data analysis, data interpretation, or writing of the report. WHO was responsible for the 2024 WHO BPPL study design, data collection, data analysis, data interpretation, and writing of the report.

## Results

The preferences survey was conducted from April 6 to May 16, 2023. Of the 100 experts invited to participate, 78 completed the survey and one completed the survey without providing full personal details; the remaining 21 incomplete responses were excluded. The participants included 24 women (30%), 54 men (70%), and 52 (66%) participants were aged 51–71 years. Geographically, 26 experts (33%) were from the WHO European Region, 23 (29%) from the Americas, ten (13%) from Africa, nine (11%) from the Western Pacific, seven (9%) from South-East Asia, and three (4%) from the Eastern Mediterranean. By income level, 46 (58%) were from high-income countries, 17 (22%) from upper-middle-income countries, and 15 (19%) from low-income or lower-middle-income countries. Professionally, 32 (41%) were clinicians, 23 (29%) microbiologists, and 24 (30%) experts in public health, epidemiology, or pharmaceuticals. Participants answered a median of 43 (IQR 33–52) pairwise comparison questions each. A strong consensus emerged on the final pathogen rankings, with a Spearman's rank correlation coefficient of 0·9 and Kendall's coefficient of concordance of 0·9. The consistency checks revealed that 50 (63%) experts answered both repeated questions correctly (consistently), 18 (23%) answered one incorrectly, and 11 (14%) answered both incorrectly (appendix p 6).

**Figure 1:**
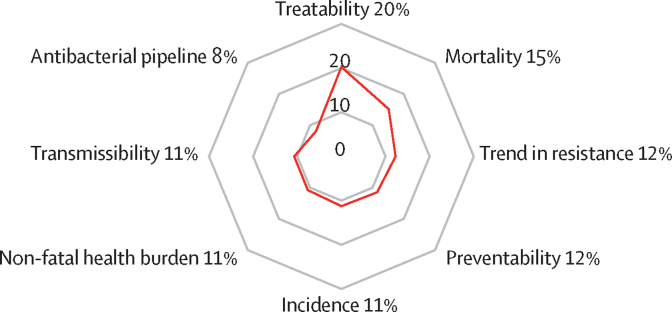
Mean criteria weights from the global preferences (PAPRIKA) survey Percentages represent the mean weights from a survey of 79 experts. Figure reproduced from 2024 WHO Bacterial Priority Pathogens List report*.*^15^ PAPRIKA=Potentially All Pairwise Rankings of All Possible Alternatives.

Figure 1 shows the mean weights assigned to each criterion based on the 79 experts who completed the preferences survey. Three criteria—treatability, mortality, and the 10-year trend in resistance—accounted for 48% of the total weight. Compared with the overall group of 78 experts (excluding one participant who did not provide personal information), experts from low-income or lower-middle-income countries assigned less weight to treatability (17% *vs* 21% overall) and pipeline (6% *vs* 8% overall), but assigned more weight to mortality (17% *vs* 16% overall) and incidence (14% *vs* 11% overall; appendix p 1). A geographical analysis mirrored these income-related differences (p=0·025 from MANOVA), particularly between the European and African regions (appendix pp 3–4).

To enhance the validity of the results, we computed the final pathogen ranking by subgrouping participants based on their expertise, geographical location, income levels, and consistency. No significant variations were observed in any subgroups or in the sensitivity analysis in which participants with fewer than one consistent response were excluded. Details on the subgroup and sensitivity analyses are available in the appendix (pp 1–6).

**Figure 2:**
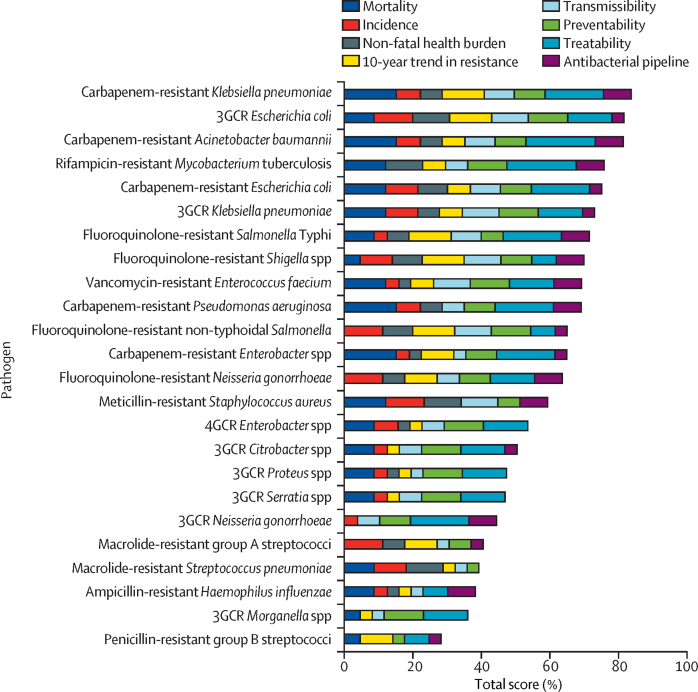
Final ranking of antibiotic-resistant bacteria in the 2024 WHO BPPL The x-axis represents the total score (0–100%) of antibiotic-resistant bacteria, with higher percentages reflecting a higher ranking of the pathogen in the 2024 WHO BPPL based on its resistance profile and public health impact. 3GCR=third-generation cephalosporin-resistant. 4GCR=fourth-generation cephalosporin-resistant. BPPL=Bacterial Priority Pathogens List. *Salmonella* Typhi=*Salmonella enterica* serotype Typhi. *Rifampicin-resistant *M tuberculosis* was included after an independent analysis with parallel criteria and subsequent application of the multicriteria decision analysis matrix.

Figure 2 presents the final ranking of antibiotic-resistant bacteria in the 2024 WHO BPPL. Total scores for the 24 pathogens ranged from 84% for carbapenem-resistant *Klebsiella pneumoniae* (top-ranked) to 28% for penicillin-resistant group B streptococci (bottom-ranked). Among Gram-positive bacteria, vancomycin-resistant *Enterococcus faecium* (69%) and meticillin-resistant *S aureus* (MRSA; 59%) were the highest ranked. Among the bacteria commonly responsible for community-acquired infections, fluoroquinolone-resistant *Salmonella enterica* serotype Typhi (*S* Typhi; 71%), *Shigella* spp (70%), and *Neisseria gonorrhoeae* (64%) ranked highest.

**Figure 3:**
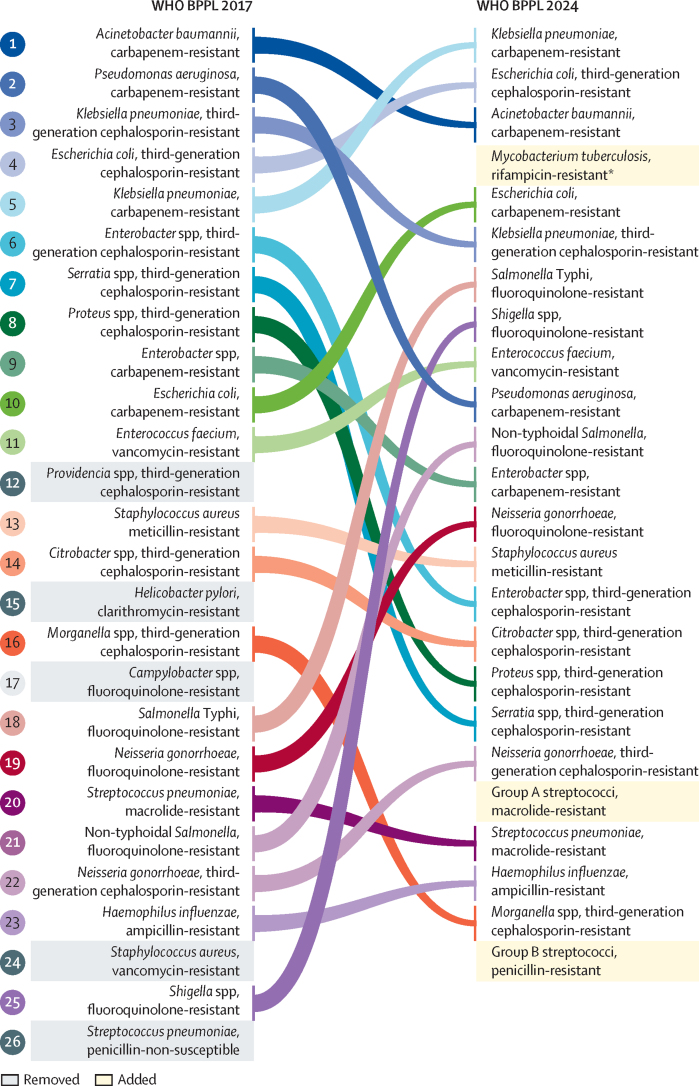
Comparative overview of WHO BPPL, 2017 versus 2024 Pathogens are ranked by position on the BPPL. Figure reproduced from the 2024 WHO BPPL report.^15^ BPPL=Bacterial Priority Pathogens List. *Salmonella* Typhi=*Salmonella enterica* serotype Typhi.*Rifampicin-resistant *M tuberculosis* was included after an independent analysis with parallel criteria and subsequent application of the multicriteria decision analysis matrix.

The final WHO BPPL 2024 list was categorised into three priority tiers: critical, high, and medium (table 2). These groupings and their interpretation were finalised with input from the WHO BPPL Advisory Group and relevant WHO programmes. Based on this categorisation, the critical tier includes carbapenem-resistant *Acinetobacter baumannii*, carbapenem-resistant and third-generation cephalosporin-resistant Enterobacterales, and rifampicin-resistant *M tuberculosis*. The high-priority tier features fluoroquinolone-resistant *S* Typhi and *Shigella* spp, vancomycin-resistant *E faecium*, carbapenem-resistant *Pseudomonas aeruginosa*, fluoroquinolone-resistant non-typhoidal *Salmonella, N gonorrhoeae* (third-generation cephalosporin resistant or fluoroquinolone resistant, or both), and MRSA. The medium-priority tier encompasses macrolide-resistant group A streptococci and *S pneumoniae*, ampicillin-resistant *Haemophilus influenzae*, and penicillin-resistant group B streptococci (table 2). Figure 3 compares the 2024 WHO BPPL with the 2017 WHO BPPL.

**Table 2:** Comparative overview of bacterial pathogen priority tiers, 2017 versus 2024

	**2017**	**2024**
Critical priority	*Acinetobacter baumannii*, carbapenem-resistant; *Pseudomonas aeruginosa*, carbapenem-resistant; Enterobacteriaceae, carbapenem-resistant, third-generation cephalosporin-resistant	*A baumannii*, carbapenem-resistant; Enterobacterales, third-generation cephalosporin-resistant; Enterobacterales, carbapenem-resistant; *Mycobacterium tuberculosis*, rifampicin-resistant*
High priority	*Enterococcus faecium*, vancomycin-resistant; *Staphylococcus aureus*, meticillin-resistant, vancomycin intermediate and-resistant; *Helicobacter pylori*, clarithromycin-resistant; *Campylobacter* spp, fluoroquinolone-resistant; salmonellae, fluoroquinolone-resistant; *Neisseria gonorrhoeae*, cephalosporin-resistant, fluoroquinolone-resistant	*Salmonella enterica* serotype Typhi, fluoroquinolone-resistant; *Shigella* spp, fluoroquinolone-resistant; *E faecium*, vancomycin-resistant; *P aeruginosa*, carbapenem-resistant; non-typhoidal *Salmonella*, fluoroquinolone-resistant; *N gonorrhoeae*, third-generation cephalosporin-resistant, fluoroquinolone-resistant; *S aureus*, meticillin-resistant
Medium priority	*Streptococcus pneumoniae*, penicillin non-susceptible; *Haemophilus influenzae*, ampicillin-resistant; *Shigella* spp, fluoroquinolone-resistant	Group A streptococci, macrolide-resistant; *S pneumoniae*, macrolide-resistant; *H influenzae*, ampicillin-resistant; group B streptococci, penicillin-resistant

Pathogens are streamlined by family or order and categorised into three priority tiers.

* Rifampicin-resistant *M tuberculosis* was included after an independent analysis with parallel criteria and subsequent application of the multicriteria decision analysis matrix.

## Discussion

The 2024 WHO BPPL is a prioritised list of bacterial pathogens, developed using current evidence and expert input through the MCDA method, to guide research and development of antibacterials and other public health interventions.^15^ The prioritisation highlights that several pathogens remain top priorities, while others have shifted in ranking, underscoring the evolving nature of the AMR threat and the need for regular evaluation of priorities.

Despite advancements in antibacterial research and development since 2017, antibiotic-resistant Gram-negative pathogens remain a critical priority. They are associated with a substantial burden globally, mostly in low-income and middle-income countries.^4^ Their diverse resistance mechanisms, ability to transfer genes horizontally, and persistence in health-care environments further complicate control efforts. Of particular concern is their increasing resistance to the small number of last-resort antibiotics.^20^ The persistence of antibiotic-resistant Gram-negative bacteria on the WHO BPPL emphasises the need for innovative and targeted research and development approaches to tackle complex resistance mechanisms, such as OXA-type β-lactamases and metallo-β-lactamases. Addressing this challenge requires overcoming considerable scientific, economic, and market barriers.^21^ Approaches should prioritise sustained research and development investments and innovative financing mechanisms to drive the development of new antimicrobials, including both push mechanisms (eg, grants, subsidies, and public–private partnerships to reduce the costs and risks associated with early-stage research and development) and pull mechanisms (eg, market entry rewards, advanced market commitments, and patent buyouts to reward successful innovations).^19^ Equally important are strengthened prevention efforts, including improvements in and equitable access to health-care systems, robust infection prevention and control measures, and vaccination. A recent analysis estimated that investing in health-care quality and the equitable introduction of new antimicrobials for Gram-negative bacteria could avert up to 92 million AMR-related deaths by 2050.^4^ A comprehensive strategy is essential for addressing the diverse needs of populations and to align with WHO's strategic and operational priorities for tackling drug-resistant bacterial infections.^22^ This approach was reinforced by the high-level political declaration on AMR at the 79th UN General Assembly in 2024, where global leaders committed to ambitious targets, including the goal to reduce AMR-related deaths by 10% by 2030, with a strong emphasis on a multidimensional response and multisectoral collaboration.^22^, ^23^

The 2024 WHO BPPL categorises third-generation cephalosporin-resistant Enterobacterales separately from carbapenem-resistant Enterobacterales to highlight their distinct challenges and need for tailored responses.^15^ Infections due to extended-spectrum β-lactamase-producing Enterobacterales are associated with a substantial burden and increased health-care costs, particularly in low-income and middle-income countries.^3^, ^4^ Bloodstream infections associated with third-generation cephalosporin-resistant Enterobacterales (ie, *Escherichia coli*) are recognised under the UN Sustainable Development Goals as part of the AMR indicator 3.d.2.^24^ In low-income settings, inadequate health-care capacity, inappropriate antibiotic use (including use of last-resort agents), and limited access to essential antibiotics and diagnostics create a feedback loop that exacerbates the burden of these pathogens and accelerates AMR.^25^ Addressing these priority pathogens requires strengthening infection prevention and control measures, improving equitable access to effective antibiotics, and enhancing stewardship programmes to break this cycle.

The 2024 WHO BPPL highlights increased prioritisation of community-acquired pathogens (ie, those acquired outside of health-care settings), reflecting newly available data and a growing recognition of their substantial burden, particularly in resource-limited settings. For example, rifampicin-resistant *M tuberculosis* is now in the critical priority category, highlighting its challenges compared with drug-susceptible *M tuberculosis*, including complexities in diagnosis, high treatment costs, and problems with drug toxicity.^26^ The emergence of *M tuberculosis* strains resistant to newer core drugs such as bedaquiline, in addition to poor access to diagnostics, highlight the urgent need for research and development investments and strengthened public health responses.^27^

Other community-acquired pathogens, such as fluoroquinolone-resistant *Salmonella* (typhoidal and non-typhoidal) and *Shigella* spp**,** have risen in the rankings in the 2024 WHO BPPL versus the 2017 version, probably due to the large disease burden and increasing resistance of these pathogens in low-income and middle-income countries.^28^, ^29^ This growing focus emphasises the need to expand AMR strategies beyond the development of new medicines to include innovations in prevention, such as vaccine development and coverage. Ensuring equitable access to quality care and investing in water, sanitation, and hygiene (WASH) infrastructure are fundamental for controlling these pathogens and communicable diseases overall. In 2021, over 2 billion people did not have access to safely managed drinking water services, and more than 4·5 billion people—over half of the global population—lacked safely managed sanitation services or were not fully covered by essential health services.^30^

Another notable update in the 2024 WHO BPPL is the reclassification of carbapenem-resistant *P aeruginosa* from critical to high.^15^ This downgrade might reflect observed decreases in resistance trends in at least one WHO region, and aligns with other estimates, such as the US Centers for Disease Control and Prevention 2019 list, which categorises carbapenem-resistant *P aeruginosa* as “serious” rather than “critical”.^31^ Although the overall burden of carbapenem-resistant *P aeruginosa* is lower than that of other critical Gram-negative pathogens, it remains an important concern in some populations and regions, particularly where resistance mechanisms are more prevalent. Therefore, reclassification to high priority does not lessen the urgency for sustained and targeted investments in research and development, which remain essential to develop effective treatments and diagnostics for carbapenem-resistant *P aeruginosa* infections.

Several pathogens, including MRSA, remain in the high priority category. Despite its considerable global burden, MRSA's ranking reflects its designation as highly treatable in the MCDA assessment.^4^ However, inequitable access to treatments and prevention measures continues to exacerbate its burden, especially in resource-limited settings, and must be urgently addressed to mitigate the threat of MRSA and reduce global disparities. Another pathogen that remains in the high priority category is vancomycin-resistant *E faecium*. While often an asymptomatic coloniser, vancomycin-resistant *E faecium* can cause severe opportunistic infections, including endocarditis and bacteraemia. The emergence of vancomycin-resistant *E faecium* is driven by multiple factors. The horizontal transfer of *vanA* and *vanB* gene clusters via plasmids facilitates the spread of resistance, and the global dissemination of clonal complex 17 (CC17) strains, which are often multidrug resistant, significantly contributes to the prevalence of vancomycin-resistant *E faecium* in health-care settings; the epidemiology is further complicated by the potential for animal-to-human transmission. Compounded by limited access to molecular diagnostics and effective treatments, these factors underscore the increasing importance of vancomycin-resistant *E faecium* as a nosocomial pathogen .^32^

Antibiotic-resistant *N gonorrhoeae* was also categorised as high priority given its increasing burden and resistance to available treatments, which pose a substantial public health threat.^33^ The burden is particularly high among women, many of whom face stigma for seeking related health care. These infections are often asymptomatic, making early detection and treatment challenging, which poses risks for complications such as infertility and facilitates the spread of the infection.^30^ Perinatal exposure to antibiotic-resistant *N gonorrhoeae* can also put neonates at risk for severe complications, such as gonococcal sepsis and blindness.^30^ Addressing antibiotic-resistant *N gonorrhoeae* requires a multifaceted approach, including innovative new medicines and prevention measures, equitable access to effective screening tools and antibiotics, and strategies to mitigate stigma, raise awareness, and establish robust monitoring systems.^30^

Other notable additions to the WHO BPPL include macrolide-resistant group A streptococci, penicillin-resistant group B streptococci, ampicillin-resistant *H influenzae*, and macrolide-resistant *S pneumoniae*. Each of these pathogens poses distinct challenges, particularly among populations at increased risk—such as young children, older adults, immunocompromised individuals, and those with underlying health conditions—especially in low-income and middle-income countries.^34^ The emergence of resistance in these organisms complicates effective treatment and control, heightening the risk of severe outcomes such as sepsis.^4^ Despite the availability of effective vaccines against some of these pathogens, regional disparities in coverage persist (eg, 83% coverage in the WHO European Region for *S pneumoniae* versus 23% in the WHO Western Pacific Region).^35^ This disparity highlights the need for improved access to and affordability of these lifesaving tools, especially in resource-limited settings.

Several important considerations should be noted regarding the WHO BPPL. The global list is not exhaustive and should be carefully contextualised to address local infection burdens, health inequities, and specific research and development gaps. While the list is primarily intended to inform research and development priorities, we recognise that addressing AMR requires a comprehensive approach. Therefore, relevant parties are encouraged to adapt the list as appropriate to guide other AMR interventions, including investments in prevention, access to medicines, surveillance, WASH, and community health infrastructure. It is important, however, to bear in mind that the assumptions underlying the list are primarily focused on research and development and should be carefully considered when applied to other areas.

Limitations of this analysis include some data gaps, particularly in regions with insufficient surveillance, which impact the assessments of mortality, incidence, and resistance trends. These gaps emphasise the need for improved global surveillance platforms powered by emerging technologies, such as artificial intelligence, which could provide real-time insights into resistance trends and disease burden, and guide data-driven interventions. The study's reliance on pooled and potentially outdated data further complicated evaluations. The prioritisation did not account for individual resistance mechanisms (or genotypes) in the final ranking and presentation of pathogens. There were some limitations in assessing transmissibility and preventability, including overlooking important nuances in transmission routes, particularly for airborne pathogens such as rifampicin-resistant *M tuberculosis*, and not considering the feasibility of applying prevention measures at the national or local levels. Due to the unique nature of rifampicin-resistant *M tuberculosis*, it was assessed through a parallel, adapted process.^15^ This included tailored criteria definitions and data sources to account for its distinct transmission pathway, chronic nature, and requirement for complex, prolonged combination therapies. For the MCDA evaluation, rifampicin-resistant *M tuberculosis* treatability was assessed solely based on WHO guidelines, while the pipeline criterion uniquely considered the need for combination therapies for rifampicin-resistant *M tuberculosis*, which might have influenced its scoring. Despite efforts to ensure gender and geographical diversity, response rates from experts in low-income and lower-middle-income countries were low, resulting in these countries being grouped together for the final analysis, and highlighting the need for continued engagement in future assessments. To minimise bias, analyses were stratified by income setting, which confirmed the stability of the final ranking.

The 2024 WHO BPPL underscores the persistent and growing challenge of AMR, with antibiotic-resistant Gram-negative bacteria and rifampicin-resistant *M tuberculosis* continuing to pose substantial threats, particularly in resource-limited settings. We call for sustained investments to develop novel antibiotics and prevention strategies. Regular updates to the list will be essential to keep pace with the evolving AMR landscape. Despite challenges, including data gaps and health inequities, the WHO BPPL remains a valuable resource for guiding research and development, policy, and health-care decisions. Overcoming scientific, economic, and market barriers, while ensuring equitable access and robust prevention measures, will be crucial to effectively address AMR.

### WHO Bacterial Priority Pathogens List Advisory Group

### Contributors

### Data sharing

The data that support the findings of this study are available from the corresponding author upon reasonable request.

## Declaration of interests

We declare no competing interests.

## Supplementary Materials

Arabic translation of the abstract

French translation of the abstract

Italian translation of the abstract

Japanese translation of the abstract

Spanish translation of the abstract
