# Supplementary material for: The WHO Bacterial Priority Pathogens List 2024: a prioritisation study to guide research, development, and public health strategies against antimicrobial resistance
Source: Lancet Infect Dis. 2025 Sep;25(9):1033–43. doi: 10.1016/S1473-3099(25)00118-5 (PMC12367593; doi:10.1016/S1473-3099(25)00118-5)
Supplement: Arabic translation of the abstract [file mmc1.pdf]

THE LANCET
Infectious Diseases

## Supplementary appendix 1

This translation in Arabic was submitted by the authors and we reproduce it as supplied. It has not been peer reviewed. *The Lancet*'s editorial processes have only been applied to the original in English, which should serve as reference for this manuscript.

تم تقديم هذه الترجمة باللغة العربية من قبل المؤلفين ونعيد إنتاجها كما هو مُقدم. إنها لم تخضع لاستعراض الأقران. تم تطبيق عمليات تحرير *لانسيت* فقط على النص الأصلي باللغة الإنجليزية، والذي يجب أن يكون بمثابة مرجع لهذه المخطوطة.

Supplement to: Sati H, Carrara E, Savoldi A, et al. The WHO Bacterial Priority Pathogens List 2024: a prioritisation study to guide research, development, and public health strategies against antimicrobial resistance. *Lancet Infect Dis* 2025; **25:** 1033–43.

**الخلفية**

كانت قائمة منظمة الصحة العالمية الصادرة عام ٢٠١٧ أداة أساسية لتوجيه السياسات نحوأولويات مسببات الأمراض البكتيرية في العالم العالمية والبحث والتطوير والاستثمارات لمعالجة أخطر التهديدات الناجمة عن مسببات الأمراض المقاومة للمضادات الحيوية. منذ صدورها ساهمت القائمة كأداة صحية عامة رئيسية للوقاية من مقاومة مضادات الميكروبات ومكافحتها. منذ صدورها، تمت الموافقة على ١٣ مضادًا حيويًا جديدًا على الأقل تستهدف مسببات الأمراض البكتيرية ذات الأولوية. تهدف الدراسة التحديثية لقائمة منظمة الصحة العالمية لاولويات مسببات الامراض البكتيرية المقاومة للمضادات الحيوية لعام٢٠٢٤ إلى تحسين القائمة السابقة وبناء عليها من خلال دمج بيانات وأدلة جديدة، ومعالجة القيود السابقة، وتحسين تحديد أولويات مسببات الأمراض لتوجيه الجهود العالمية بشكل أفضل في مكافحة مقاومة مضادات الميكروبات.

**المنهجية**

اتبعت قائمة منظمة الصحة العالمية لعام ٢٠٢٤ منهجًا مشابهًا للدراسة الأولي التي تمت في عام ٢٠١٧، باستخدام إطار تحليل قرار متعدد المعايير. تم تقييم ٢٤ من مسببات الأمراض البكتيرية المقاومة للمضادات الحيوية بناءً على ثمانية معايير تشمل الوفيات، والعبء غير المميت، والوقوع، واتجاهات تغيرالمقاومة على مدى ١٠ سنوات، والقابلية للوقاية، والقابلية للانتقال، والقابلية للعلاج، وحالة برامج المضادات الحيوية قيد التطوير. تم تقييم مسببات الأمراض بناءً على كل معيار باستخدام الأدلة المتاحة وحكم الخبراء. تم إجراء استبيان تفضيلات باستخدام المقارنة الزوجية مع ٧٩ خبيرًا دوليًا لتحديد الأوزان النسبية للمعايير. بتطبيق هذه الأوزان، تم تحديد التصنيف النهائي لمسببات الأمراض عن طريق حساب درجة إجمالية تتراوح بين 0-100٪ لكل مسبب. تم إجراء تحليلات فرعية وحساسية لتقييم تأثير اتساق الخبراء وخلفياتهم وأصولهم الجغرافية على استقرار التصنيفات. قامت مجموعة استشارية مستقلة بمراجعة القائمة النهائية، وتم تبسيط مسببات الأمراض وتجميعها في ثلاث مستويات أولوية بناءً على نظام تصنيف رباعي: حرج (الربع الأعلى)، عالي (الأرباع الوسطى)، ومتوسط (الربع الأدنى).

**النتائج**

تراوحت الدرجات الإجمالية لمسببات الأمراض من ٨٤٪ لأعلى بكتيريا مصنفة (المكورات الرئوية الكلبسيلية المقاومة للكاربابينيم) إلى ٢٨٪ لأدنى بكتيريا مصنفة (العقديات من المجموعة ب المقاومة للبنسلين). احتلت البكتيريا سلبية الغرام المقاومة للمضادات الحيوية (بما في ذلك الكلبسيلا الرئوية، الأسينتوباكتر، والإشريكية القولونية)، بالإضافة إلى المتفطرة السلية المقاومة للريفامبيسين، الربع الأعلى. ومن بين البكتيريا المسؤولة عادةً عن التهابات المجتمع المكتسبة، كانت أعلى التصنيفات للسالمونيلا التيفية المقاومة للفلوروكينولون (٧٢٪)، الشيغيلا (٧٠٪)، والمكورات البنية (٦٤٪). تضمنت القائمة مسببات أمراض مهمة أخرى مثل الزائفة الزنجارية والمكورات العنقودية الذهبية. أظهرت نتائج استبيان التفضيلات اتفاقًا قويًا بين االخبراء، حيث كان معامل ارتباط سبيرمان ومعامل كيندال للاتفاق كلاهما عند 0.9. وأظهر التصنيف النهائي استقرارًا عاليًا، حيث أن تجميع مسببات الأمراض بناءً على خلفية الخبراء وأصلهم لم يؤد إلى أي تغييرات جوهرية في التصنيف.

**الاستنتاجات**

تمثل قائمة منظمة الصحة العالمية لعام ٢٠٢٤ أداة رئيسية لتحديد أولويات استثمارات البحث والتطوير وإعلام السياسات الصحية العامة العالمية لمكافحة مقاومة مضادات الميكروبات. تظل البكتيريا سلبية الغرام والمتفطرة السلية المقاومة للريفامبيسين مسببات أمراض ذات أولوية حرجة، مما يؤكد تهديدها المستمر وعدم كفاية الاسثمارات الحالية لانتاج مضادات حيوية جديدة وفعالة . هناك حاجة إلى جهود مركزة واستثمارات مستدامة في مضادات بكتيرية جديدة لمعالجة مسببات الأمراض ذات الأولوية لمقاومة مضادات الميكروبات، والتي تشمل بكتيريا مقاومة للمضادات الحيوية ذات عبء صحي مرتفع مثل السالمونيلا والشيغيلا، والمكورات البنية، والمكورات العنقودية الذهبية. إلى جانب البحث والتطوير، يجب أن

تشمل الجهود لمعالجة هذه المسببات أيضًا توسيع الوصول العادل إلى الأدوية الحالية، وتعزيز تغطية اللقاحات، وتدابير الوقاية من العدوى ومكافحتها.
