# Supplementary material for: The WHO Bacterial Priority Pathogens List 2024: a prioritisation study to guide research, development, and public health strategies against antimicrobial resistance
Source: Lancet Infect Dis. 2025 Sep;25(9):1033–43. doi: 10.1016/S1473-3099(25)00118-5 (PMC12367593; doi:10.1016/S1473-3099(25)00118-5)
Supplement: French translation of the abstract [file mmc2.pdf]

THE LANCET
Infectious Diseases

## Supplementary appendix 2

This translation in French was submitted by the authors and we reproduce it as supplied. It has not been peer reviewed. *The Lancet*'s editorial processes have only been applied to the original in English, which should serve as reference for this manuscript.

Cette traduction en français a été proposée par les auteurs et nous l'avons reproduite telle quelle. Elle n'a pas été examinée par des pairs. Les processus éditoriaux du *Lancet* n'ont été appliqués qu'à l'original en anglais et c'est cette version qui doit servir de référence pour ce manuscrit.

Supplement to: Sati H, Carrara E, Savoldi A, et al. The WHO Bacterial Priority Pathogens List 2024: a prioritisation study to guide research, development, and public health strategies against antimicrobial resistance. *Lancet Infect Dis* 2025; **25:** 1033–43.

## Résumé

**Contexte** La Liste des agents pathogènes bactériens prioritaires de l'OMS (BPPL) de 2017 a joué un rôle clé dans l'orientation des politiques mondiales, de la recherche et du développement, ainsi que des investissements pour faire face aux menaces les plus urgentes posées par les agents pathogènes résistants aux antibiotiques. Elle constitue un outil essentiel de santé publique pour la prévention et le contrôle de la résistance aux antimicrobiens (RAM). Depuis sa publication, au moins 13 nouveaux antibiotiques ciblant les agents pathogènes bactériens prioritaires ont été approuvés. La BPPL 2024 de l'OMS vise à affiner et à développer la liste précédente en intégrant de nouvelles données et preuves, en présentant les limites précédemment observées et en améliorant la hiérarchisation des agents pathogènes pour mieux guider les efforts mondiaux dans la lutte contre la RAM.

## Méthodes

La BPPL 2024 de l'OMS a suivi une approche similaire à la première hiérarchisation, en utilisant un cadre d'analyse décisionnelle multicritère. Vingt-quatre agents pathogènes bactériens résistants aux antibiotiques ont été évalués sur la base de huit critères, notamment la mortalité, la charge non mortelle, l'incidence, les tendances de résistance sur 10 ans, la préventabilité, la transmissibilité, la traîtabilité et l'état du pipeline d'antibactériens. Les agents pathogènes ont été évalués pour chaque critère sur la base des preuves disponibles et du jugement d'experts. Une enquête de préférences utilisant une comparaison par paires a été menée auprès de 79 experts internationaux pour déterminer les poids relatifs des critères. En appliquant ces poids, le classement final des agents pathogènes a été déterminé en calculant un score total compris entre 0 et 100 % pour chaque agent pathogène. Des analyses de sous-groupes et de sensibilité ont été réalisées pour évaluer l'impact de la cohérence, du profil et de l'origine géographique des experts sur la stabilité des classements. Un groupe consultatif indépendant a examiné la liste finale, celle-ci a été affinée, puis  les agents pathogènes ont ensuite été regroupés en trois niveaux de priorité selon un système de notation par quartiles : critique (quartile supérieur), élevé (quartiles intermédiaires) et moyen (quartile inférieur).

## Résultats

Les scores totaux des agents pathogènes variaient de 84 % pour la bactérie la mieux classée (*Klebsiella pneumoniae* résistante aux carbapénèmes) à 28 % pour la bactérie la moins bien classée (streptocoques du groupe B résistants à la pénicilline). Les bactéries à Gram négatif résistantes aux antibiotiques (y compris *K. pneumoniae*, *Acinetobacter spp.* et *Escherichia coli*), ainsi que *Mycobacterium tuberculosis* résistant à la rifampicine, ont été

classées dans le quartile supérieur. Parmi les bactéries fréquemment responsables d'infections communautaires, les classements les plus élevés étaient *Salmonella enterica* sérovar Typhi résistante aux fluoroquinolones (72 %), *Shigella spp* (70 %) et *Neisseria gonorrhoeae* (64 %). D'autres agents pathogènes importants ont été inclus dans la liste à savoir *Pseudomonas aeruginosa* et *Staphylococcus aureus*. Les résultats de l'enquête de préférences ont montré un fort accord inter-évaluateurs, avec un coefficient de corrélation de Spearman et un coefficient de concordance de Kendall tous deux à 0,9. Le classement final a montré une grande stabilité, le regroupement des agents pathogènes en fonction du profil et de l'origine des experts n'entraînant aucun changement substantiel dans le classement.

### Interprétation

La BPPL 2024 de l'OMS est un outil clé pour hiérarchiser les investissements en recherche et développement et éclairer les politiques de santé publique mondiales dans la lutte contre la RAM. Les bactéries à Gram négatif et *M. tuberculosis* résistant à la rifampicine restent des agents pathogènes prioritaires critiques, soulignant leur menace persistante et les limites du pipeline actuel d'antibactériens. Des efforts ciblés et des investissements soutenus dans de nouveaux antibactériens sont nécessaires pour lutter contre les agents pathogènes prioritaires de la RAM, qui incluent des bactéries résistantes aux antibiotiques à forte charge telles que *Salmonella* et *Shigella spp.*, *N. gonorrhoeae* et *S. aureus*. Au-delà de la recherche et du développement, les efforts pour lutter contre ces agents pathogènes devraient également inclure l'élargissement de l'accès équitable aux médicaments existants, l'amélioration de la couverture vaccinale et le renforcement des mesures de prévention et de contrôle des infections.
