# Supplementary material for: The WHO Bacterial Priority Pathogens List 2024: a prioritisation study to guide research, development, and public health strategies against antimicrobial resistance
Source: Lancet Infect Dis. 2025 Sep;25(9):1033–43. doi: 10.1016/S1473-3099(25)00118-5 (PMC12367593; doi:10.1016/S1473-3099(25)00118-5)
Supplement: Italian translation of the abstract [file mmc3.pdf]

THE LANCET
Infectious Diseases

Supplementary appendix 3

This translation in Italian was submitted by the authors and we reproduce it as supplied. It has not been peer reviewed. *The Lancet*'s editorial processes have only been applied to the original in English, which should serve as reference for this manuscript.

Questa traduzione in italiano è stata proposta dagli autori ed è riportata senza modifiche. Il testo tradotto in italiano non è stato sottoposto al processo di revisione paritaria. Il processo editoriale del *Lancet* è stato esclusivamente adottato per l'originale in inglese, che dovrebbe servire da riferimento per questo articolo.

Supplement to: Sati H, Carrara E, Savoldi A, et al. The WHO Bacterial Priority Pathogens List 2024: a prioritisation study to guide research, development, and public health strategies against antimicrobial resistance. *Lancet Infect Dis* 2025; **25:** 1033–43.

Sommario

Contesto

La Lista dei patogeni batterici prioritari dell'OMS (BPPL) del 2017 ha rappresentato uno strumento fondamentale per orientare le politiche globali in materia di ricerca, sviluppo e investimenti, al fine di contrastare le minacce più urgenti poste dalla resistenza antimicrobica (AMR). Dalla pubblicazione della BPPL 2017, almeno 13 nuovi antibiotici con attività sui patogeni batterici prioritari sono stati approvati. La BPPL 2024 mira ad aggiornare e migliorare quella precedente, integrando nuove evidenze e superando le limitazioni emerse, con l'obiettivo di rafforzare la risposta globale all'AMR.

Metodi

La BPPL 2024 dell'OMS ha seguito un approccio simile al precedente esercizio di prioritizzazione, basato su una metodologia di Analisi Multicriterio. Ventiquattro patogeni batterici resistenti agli antibiotici sono stati valutati in base a otto criteri: mortalità, morbidità, incidenza, *trend* della resistenza, disponibilità di misure di prevenzione, trasmissibilità, disponibilità di terapie e stato della pipeline degli antibiotici. I patogeni sono stati classificati per ciascun criterio sulla base delle evidenze disponibili e del parere dell'Advisory Group.

Settantanove esperti sono stati quindi coinvolti in una survey, basata sul confronto iterativo tra due potenziali patogeni, al fine di computare sulla base delle preferenze indicate, i relativi pesi dei criteri. Applicando questi pesi, è stata finalizzata la classifica dei patogeni, attribuendo a ciascuno di essi un punteggio compreso tra 0 e 100%.

La validità della classifica è stata successivamente testata tramite analisi di sottogruppo e di sensibilità, stratificando i risultati in base alla provenienza geografica degli esperti, al loro *background* scientifico e alla coerenza fornita nelle risposte. La lista finale è stata infine revisionata dall'Advisory Group, e i patogeni sono stati raggruppati in tre livelli di priorità, basati su un sistema di punteggio per quartili: critico (quartile superiore), alto (quartili intermedi) e medio (quartile inferiore).

Risultati

I punteggi totali attribuiti ai patogeni hanno mostrato una variabilità compresa tra l'84% per il batterio in prima posizione (*Klebsiella pneumoniae* resistente ai carbapenemi) e il 28% per il batterio in ultima posizione (Streptococchi di gruppo B resistenti alla penicillina). I batteri Gram-negativi resistenti agli antibiotici (inclusi *K. pneumoniae*, *Acinetobacter spp*. ed *Escherichia coli*), così come *Mycobacterium tuberculosis* resistente alla rifampicina, sono stati classificati nel quartile superiore.

Tra i batteri comunemente responsabili di infezioni acquisite in comunità, i patogeni con più elevato punteggio sono: *Salmonella enterica* sierotipo Typhi resistente ai fluorochinoloni (72%), *Shigella spp*. (70%) e *Neisseria gonorrhoeae* (64%). Anche *Pseudomonas aeruginosa* e *Staphylococcus aureus* sono emersi come patogeni di rilevanza prioritaria.

La survey ha evidenziato un elevato grado di accordo tra gli esperti, con coefficienti di correlazione di Spearman e di concordanza di Kendall pari a 0,9. La classifica finale ha mostrato un'elevata stabilità, senza modifiche significative evidenziate dalle analisi di sottogruppo e di sensibilità.

Interpretazione

La BPPL 2024 dell'OMS rappresenta un riferimento strategico per orientare gli investimenti in ricerca e sviluppo e per supportare le politiche globali di sanità pubblica nella lotta all'AMR. I batteri Gram-negativi e *M. tuberculosis* resistente alla rifampicina restano patogeni di massima priorità, a testimonianza della minaccia continua che rappresentano, della carenza di terapie efficaci e dei limiti dell'attuale pipeline degli antibiotici.

È essenziale rafforzare gli investimenti nella scoperta di nuovi antibiotici, soprattutto contro i patogeni che causano infezioni comunitarie ad alta incidenza, come *Salmonella spp*., *Shigella spp*., *N. gonorrhoeae* e *S. aureus*. Tuttavia, oltre allo sviluppo di nuovi farmaci, è fondamentale migliorare l'equità nell'accesso ai trattamenti esistenti, potenziare la copertura vaccinale e rafforzare le misure di prevenzione e controllo delle infezioni.
