# Supplementary material for: The WHO Bacterial Priority Pathogens List 2024: a prioritisation study to guide research, development, and public health strategies against antimicrobial resistance
Source: Lancet Infect Dis. 2025 Sep;25(9):1033–43. doi: 10.1016/S1473-3099(25)00118-5 (PMC12367593; doi:10.1016/S1473-3099(25)00118-5)
Supplement: Japanese translation of the abstract [file mmc4.pdf]

THE LANCET
Infectious Diseases

Supplementary appendix 4

This translation in Japanese was submitted by the authors and we reproduce it as supplied. It has not been peer reviewed. *The Lancet*'s editorial processes have only been applied to the original in English, which should serve as reference for this manuscript.

この日本語 による翻訳文書は、著者本人により提出されたものを当方で複製したものです。本訳文の内容は査読を経ておらず、『ランセット』誌による編集も、本訳文のもとになる英語原文に対してのみ行われていることをご了承ください。

Supplement to: Sati H, Carrara E, Savoldi A, et al. The WHO Bacterial Priority Pathogens List 2024: a prioritisation study to guide research, development, and public health strategies against antimicrobial resistance. *Lancet Infect Dis* 2025; **25:** 1033–43.

要約

背景

2017 年の WHO 細菌優先病原体リスト（BPPL）は、抗生物質耐性病原体による最も緊急の脅威に対処するための世界的な政策、研究開発、および投資に重要な役割を果たしており、抗微生物薬耐性（AMR）の予防と制御のための重要な公衆衛生ツールです。このリストが公開されて以来、少なくとも 13 種類の新しい抗生物質が優先的な病原細菌を標的として承認されています。2024 年の WHO BPPL は、前回のリストを改善・強化することを目的としており、新たなデータや証拠を取り入れ、以前の制約に対処し、病原体の優先順位付けを向上させることで、AMR に対する世界的な取り組みをより効果的に導くことを目指しています。

方法

2024 年の WHO BPPL は、最初の優先順位付けと同様のアプローチを採用し、複数基準意思決定分析フレームワークを使用しました。24 種類の抗生物質耐性病原細菌は、死亡率、非致死的負荷、発生率、10 年間の耐性傾向、予防可能性、伝播性、治療可能性、抗菌薬パイプラインの準備状況を含む 8 つの基準に基づいて評価されました。病原体は、利用可能なエビデンスと専門家の判断に基づいて各基準で評価されました。また、基準の相対的重みを決定するために、79 人の国際的な専門家を対象にペアワイズ比較を用いた優先順位アンケートが実施されました。この重みづけを適用して、病原体の最終的な順位は、各病原体を 0～100％の範囲で総得点を計算することによって決定されました。またサブグループ分析と感度分析が実施され、専門家の整合性、背景、地理的な出身が順位の安定性に与える影響が評価されました。最終リストは独立した諮問グループによってレビューされ、その後、病原体は四分位スコアリングシステムに基づいて 3 つの優先順位層（最重要（上位四分位）、高（中間四分位）、中（下位四分位）に整理されました。

結果

病原体の総合スコアは、最も高いランクに位置する細菌（カルバペネム耐性肺炎桿菌）の 84%から最も低いランクに位置する細菌（ペニシリン耐性 B 群連鎖球菌）の 28%までの範囲でした。抗生物質耐性のグラム陰性菌（肺炎桿菌、アシネトバクター属菌、大腸菌を含む）やリファンピシン耐性結核菌は、最上位四分位にランクインしました。一般的に市中感染を引き起こす細菌の中では、最も高いランクに位置したのはフルオロキノロン耐性サルモネラ・エンテリカ血清型チフス（72%）、赤痢菌属（70%）、淋菌（64%）でした。リストに含まれる他の重要な病原体には、緑膿菌や黄色ブドウ球菌が含まれます。優先順位アンケートの結果は、評価者間で強い一致を示しており、スピアマンの順位相関係数とケンドールの一致係数はどちらも 0.9 でした。最終的な順位は高い安定性を示し、専門家の背景や出身地域に基づいて病原体をグループ化しても、順位に大きな変化はありませんでした。

解釈

2024 年の WHO BPPL は、抗微生物薬耐性（AMR）への対策として、研究開発投資の優先順位付けや、世界的な公衆衛生政策の策定のための重要なツールです。グラム陰性菌とリファンピシン耐性結核菌は依然として重要な優先病原体であり、これらが引き続き脅威であることと、現在の抗菌薬パイプラインの限界を強調しています。サルモネラや赤痢菌属、淋菌、黄色ブドウ球菌などの高負荷の抗生物質耐性菌を対象とした新規抗菌薬への集中的な取り組みと持続的な投資が必要です。研究開発に加え、これらの病原体への対策には、既存薬への公平なアクセスの拡大、ワクチン接種率の向上、感染予防・制御の強化も含まれるべきです。
