# Supplementary material for: The WHO Bacterial Priority Pathogens List 2024: a prioritisation study to guide research, development, and public health strategies against antimicrobial resistance
Source: Lancet Infect Dis. 2025 Sep;25(9):1033–43. doi: 10.1016/S1473-3099(25)00118-5 (PMC12367593; doi:10.1016/S1473-3099(25)00118-5)
Supplement: Spanish translation of the abstract [file mmc5.pdf]

THE LANCET
Infectious Diseases

Supplementary appendix 5

This translation in Spanish was submitted by the authors and we reproduce it as supplied. It has not been peer reviewed. *The Lancet*'s editorial processes have only been applied to the original in English, which should serve as reference for this manuscript.

Los autores nos proporcionaron esta traducción al español y la reproducimos tal como nos fue entregada. No la hemos revisado. Los procesos editoriales de *The Lancet* se han aplicado únicamente al original en inglés, que debe servir de referencia para este manuscrito.

Supplement to: Sati H, Carrara E, Savoldi A, et al. The WHO Bacterial Priority Pathogens List 2024: a prioritisation study to guide research, development, and public health strategies against antimicrobial resistance. *Lancet Infect Dis* 2025; **25:** 1033–43.

Resumen

Antecedentes

La Lista de Patógenos Bacterianos Prioritarios de la OMS (BPPL) de 2017 ha sido fundamental para guiar políticas globales, investigación y desarrollo, e inversiones para abordar las amenazas más urgentes de patógenos resistentes a los antibióticos, y es una herramienta clave de salud pública para la prevención y el control de la resistencia a los antimicrobianos (RAM). Desde su publicación, se han aprobado al menos 13 nuevos antibióticos dirigidos a patógenos bacterianos prioritarios. La BPPL 2024 de la OMS tiene como objetivo refinar y ampliar la lista anterior incorporando nuevos datos y evidencias, abordando limitaciones previas y mejorando la priorización de patógenos para guiar mejor los esfuerzos globales contra la RAM.

Métodos

La BPPL 2024 de la OMS siguió un enfoque similar al primer ejercicio de priorización, utilizando un marco de análisis de decisiones multicriterio. Se evaluaron 24 patógenos bacterianos resistentes a antibióticos basados en ocho criterios, incluyendo mortalidad, carga no mortal, incidencia, tendencias de resistencia a 10 años, prevención , transmission , tratamiento y estado de la cartera de antibacterianos. Los patógenos fueron evaluados para cada criterio basándose en evidencias disponibles y juicio de expertos. Se realizó una encuesta de preferencias mediante comparación por pares con 79 expertos internacionales para determinar los pesos relativos de los criterios. Aplicando estos pesos, la clasificación final de patógenos se determinó calculando una puntuación total entre 0-100% para cada patógeno. Se realizaron análisis de subgrupos y sensibilidad para evaluar el impacto de la consistencia, perfil y origen geográfico de los expertos en la estabilidad de las clasificaciones. Un grupo asesor independiente revisó la lista final, y posteriormente los patógenos se simplificaron y agruparon en tres niveles de prioridad según un sistema de puntuación por cuartiles: crítico (cuartil superior), alto (cuartil intermedio) y medio (cuartil inferior).

Resultados

Las puntuaciones totales de los patógenos oscilaron entre 84% para la bacteria de mayor clasificación (*Klebsiella pneumoniae* resistente a carbapenémicos) y 28% para la bacteria de menor clasificación (*Streptococcus* del grupo B resistentes a penicilina). Las bacterias gramnegativas resistentes a antibióticos (incluyendo *K. pneumoniae*, *Acinetobacter* spp. y

Escherichia coli), así como *Mycobacterium tuberculosis* resistente a rifampicina, se situaron en el cuartil superior. Entre las bacterias comúnmente responsables de infecciones comunitarias, las clasificaciones más altas fueron para *Salmonella enterica* serotipo Typhi resistente a fluoroquinolonas (72%), *Shigella* spp. (70%) y *Neisseria gonorrhoeae* (64%). Otros patógenos importantes en la lista incluyen *Pseudomonas aeruginosa* y *Staphylococcus aureus*. Los resultados de la encuesta de preferencias mostraron un fuerte acuerdo entre evaluadores, con coeficientes de correlación de Spearman y concordancia de Kendall ambos en 0,9. La clasificación final mostró alta estabilidad, sin cambios sustanciales al agrupar patógenos según perfil y origen de los expertos.

Interpretación

La BPPL 2024 de la OMS es una herramienta clave para priorizar inversiones en I+D e informar políticas globales de salud pública contra la RAM. Las bacterias gramnegativas y *M. tuberculosis* resistente a rifampicina siguen siendo patógenos prioritarios críticos, destacando su amenaza persistente y las limitaciones de la cartera actual de antibacterianos. Se necesitan esfuerzos focalizados e inversiones sostenidas en nuevos antibacterianos para abordar patógenos prioritarios de RAM, incluyendo bacterias resistentes de alta carga como *Salmonella*/*Shigella* spp, *N. gonorrhoeae* y *S. aureus*. Más allá de I+D, las estrategias deben incluir acceso equitativo a fármacos existentes, mayor cobertura vacunal y fortalecimiento de las medidas de prevención y control de infecciones.
