# Supplementary material for: The WHO Bacterial Priority Pathogens List 2024: a prioritisation study to guide research, development, and public health strategies against antimicrobial resistance
Source: Lancet Infect Dis. 2025 Sep;25(9):1033–43. doi: 10.1016/S1473-3099(25)00118-5 (PMC12367593; doi:10.1016/S1473-3099(25)00118-5)

THE LANCET
Infectious Diseases

Supplementary appendix 6

This appendix formed part of the original submission and has been peer reviewed. We post it as supplied by the authors.

Supplement to: Sati H, Carrara E, Savoldi A, et al. The WHO Bacterial Priority Pathogens List 2024: a prioritisation study to guide research, development, and public health strategies against antimicrobial resistance. *Lancet Infect Dis* 2025; **25:** 1033–43.

Subgroup analysis of the criteria by Income, Geography and Scientific Background

Table S1 Criteria weights stratified by income

Income*	Treatability	Mortality	Trend	Incidence	Preventability	YLDs	Transmissibility	Pipeline
**Overall**	**20.7%**	**15.7%**	**11.9%**	**11.3%**	**11.2%**	**11.1%**	**10.5%**	**7.8%**
**High-Income Countries**	21.9%	14.9%	11.5%	10.1%	10.9%	10.8%	10.7%	9.1%
**Upper-Middle Income Countries**	**18.0%**	13.9%	13.8%	12.1%	12.6%	11.5%	10.7%	7.4%
**Low- Middle Income, and Low-Income Countries (grouped in the analyses)**	**17.1%**	**17.4%**	12.9%	**14.2%**	11.9%	10.4%	10.2%	**5.8%**

**Variations higher than 1.5% are highlighted in bold¨*

Table S2 Criteria weights stratified by geography.

Region*	Treatability	Mortality	Trend	Incidence	Preventability	YLD	Transmissibility	Pipeline
**Overall**	**20.7%**	**15.7%**	**11.9%**	**11.3%**	**11.2%**	**11.1%**	**10.5%**	**7.8%**
African Region (AFR)	**15.3%**	17.2%	13.1%	**14.0%**	13.2%	11.1%	9.6%	6.4%
Region of the Americas (AMR)	18.7%	14.5%	**14.8%**	11.0%	10.8%	11.6%	11.7%	7.0%
Eastern Mediterranean Region (EMR)	**17.5%**	15.5%	**16.8%**	**15.0%**	8.6%	8.3%	12.3%	6.0%
European Region (EUR)	**24.0%**	14.8%	10.0%	10.8%	10.5%	10.2%	10.5%	9.0%
South-East Asian Region (SEAR)	18.8%	18.0%	12.6%	10.9%	11.4%	10.1%	9.5%	8.7%
Western Pacific Region (WPR)	20.1%	13.6%	9.6%	9.7%	**15.1%**	11.8%	9.6%	**10.5%**

**Variations higher than 2.5% are highlighted in bold*

Table S3 Criteria weights stratified by experts' background

Experts' background*	Treatability	Mortality	Trend	Preventability	Incidence	YLD	Transmissibility	Pipeline
**Overall**	**20.7%**	**15.7%**	**11.9%**	**11.3%**	**11.2%**	**11.1 %**	**10.5%**	**7.8%**
**Clinician**	20.2%	15.3%	12.3%	9.9%	12.2%	11.6 %	9.8%	8.7%
**Microbiologist**	20.6%	16.4%	12.1%	11.1%	11.5%	**9.5%**	11.5%	7.3%
**Public Health & Other**	20.0%	**13.7%**	12.5%	**13.9%**	9.7%	11.0 %	11.1%	8.2%

**Variations higher than 1.5% are highlighted in bold*

The Multivariate Analysis of Variance (MANOVA) was applied, given the presence of more than one dependent variable (in this case, the outcomes were represented by the 8 weights of the criteria). The covariate was represented by groups, including 3 income classes—High, Upper-Middle, and Low and LMIC (the latter two were combined due to the limited representation of Low-income countries)—and 6 WHO Regions. The assumption of multivariate normality, required for the MANOVA, was preliminarily checked through the Doornik-Hansen test (p = 0.242, non-significant, i.e. multivariate normality could be assumed).

Applying the MANOVA on all the 8 single criteria yielded no significant differences, neither in income- nor in WHO Region-based group comparisons (p = 0.714 and p = 0.742, respectively). However, the absence of significant differences could be reasonably due, to a certain extent, to the high number of groups and variables considered, in relation to the actual number of received responses. Hence, a further MANOVA was performed, considering only two parameters as dependent variables: the combined weight of drug therapy-related criteria (treatability + pipeline) and the cumulative weight of all the 6 other criteria: here, at least one significant difference was seen both by income class (p=0.002) and by WHO Region (p=0.025).

Tukey's differences were evaluated to detect where significant differences could be noticed: significant differences were found between High and Low/Low-Middle income (p = 0.028) and, as for WHO Regions, a gradient was seen - mainly due to income distribution in the respective geographic areas - with a significant difference between Europe and Africa (p = 0.044).

Table S4 Multivariate Analysis of Variance (MANOVA) by income and geography

Income comparison	*Difference [95% CI]*	*Adjusted p-value*
High vs. Low- and LMIC	0.081 [0.007; 0.154]	0.028*
High vs. Upper-Middle Income Countries (Upper-MICs)	0.056 [-0.014; 0.126]	0.139
Upper-Middle Income Countries (Upper-MICs) vs. Low- and LMIC	0.024 [-0.063; 0.112]	0.784
**Region comparison**	**Difference [95% CI]**	**Adjusted p-value**
EUR - AFR	0.114 [0.002; 0.225]	0.044*
EUR - EMR	0.096 [-0.087; 0.279]	0.643
EUR - AMR	0.074 [-0.012; 0.160]	0.133
EUR - SEAR	0.056 [-0.072; 0.184]	0.793
EUR - WPR	0.025 [-0.092; 0.141]	0.989
WPR - AFR	0.089 [-0.049; 0.227]	0.419
WPR - EMR	0.071 [-0.129; 0.272]	0.902
WPR - AMR	0.049 [-0.069; 0.167]	0.825
WPR - SEAR	0.031 [-0.120; 0.183]	0.990
SEAR - AFR	0.058 [-0.091; 0.206]	0.864
SEAR - EMR	0.040 [-0.167; 0.247]	0.993
SEAR - AMR	0.018 [-0.112; 0.148]	0.999
AMR - EMR	0.040 [-0.074; 0.153]	0.910
AMR - AFR	0.022 [-0.162; 0.207]	0.999
EMR - AFR	0.018 [-0.180; 0.215]	1.000

Figure S1 Ranking variation by participants' consistency.

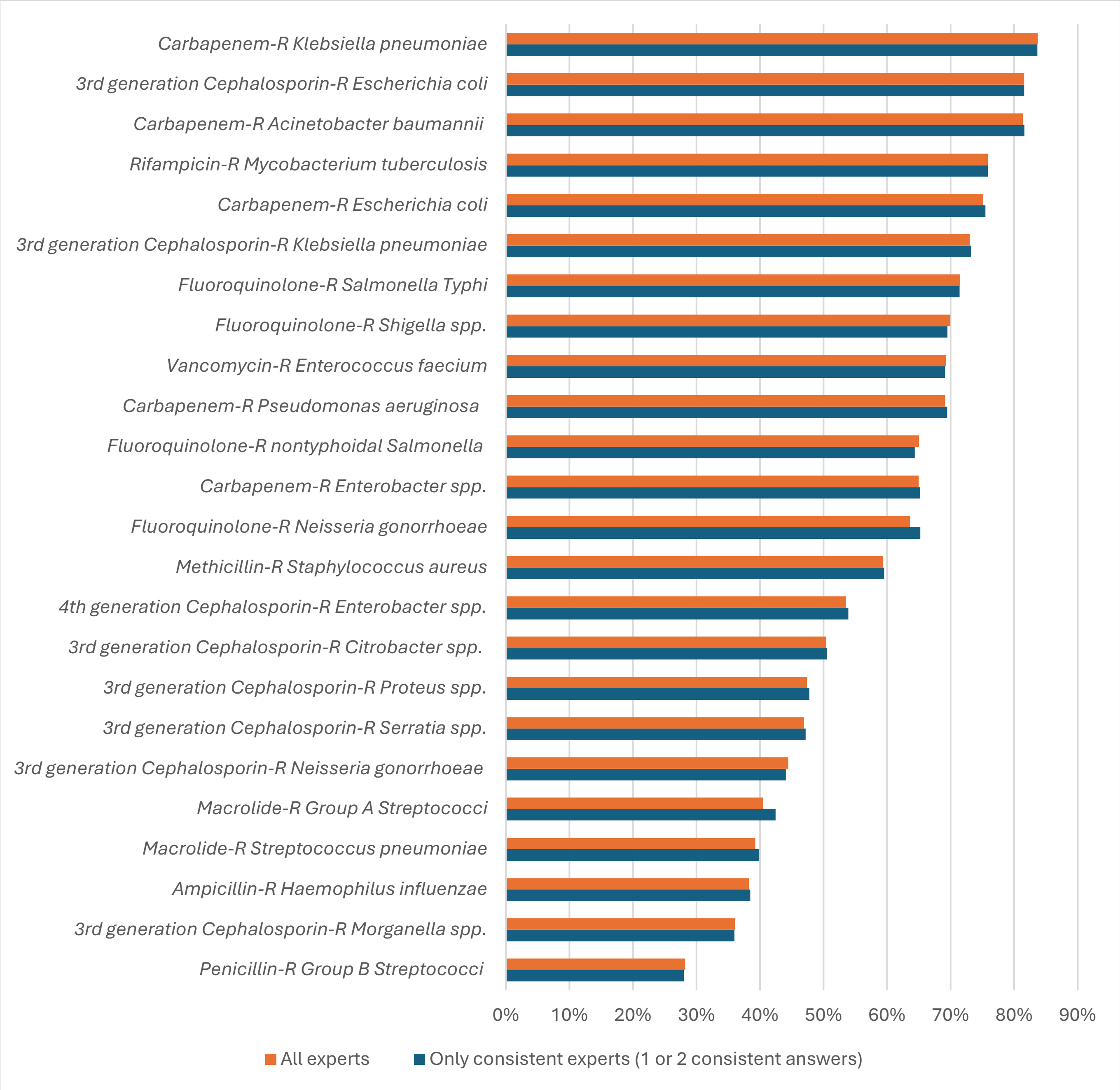

Figure S2 Expert's consistency (number of correct repeated questions)

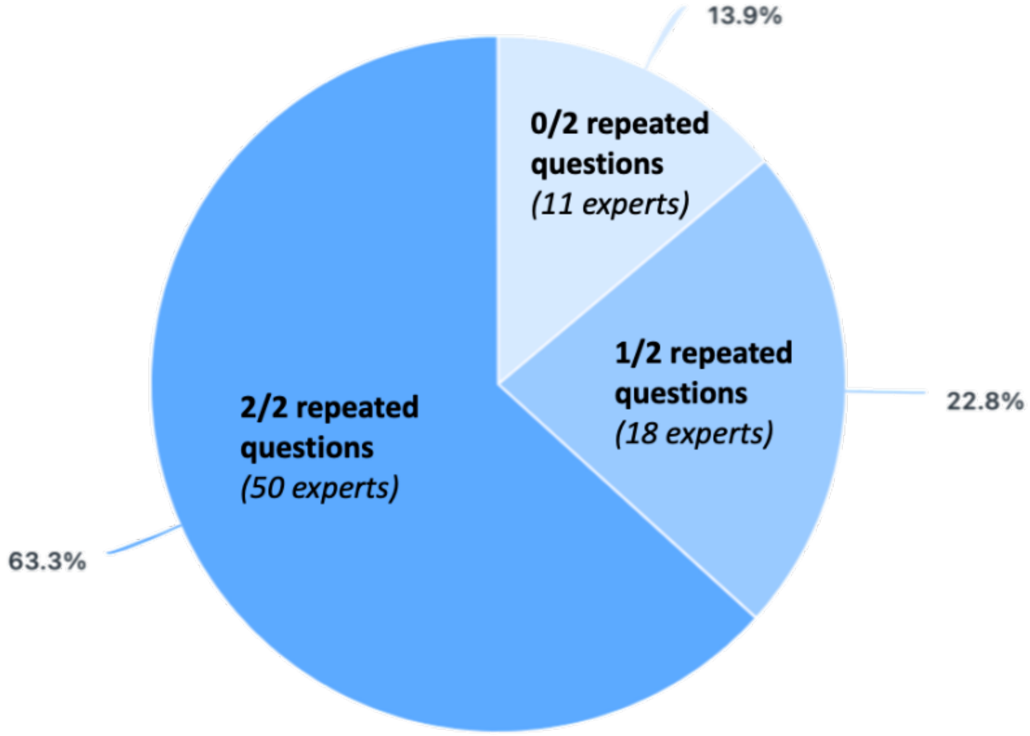

Supplement: Supplementary appendix 6 [file mmc6.pdf]
